# Intravitreal injection technique

**Published:** 2014

**Authors:** David Yorston

**Affiliations:** Consultant Ophthalmologist, Tennent Institute of Ophthalmology, Gartnavel Hospital, Glasgow, UK dbyorston@btinternet.com

**Figure:**
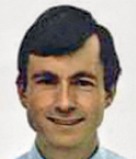
David Yorston

This article describes how to give an intravitreal injection of an anti-VEGF drug.

At present, the majority of intravitreal injections are administered by doctors; however, experience in the UK has shown that trained nurses can give these injections as safely as ophthalmologists.

The single most important thing to remember is that an intravitreal injection is an intraocular operation, and should be treated equally seriously. The most devastating complication is endophthalmitis, and you must take every possible precaution to prevent infection.

## Setting

Intravitreal injections should be given in a clean room. A clean room is a room dedicated to intraocular injections or other sterile interventions. The most important point is that a clean room should never be used for unsterile or dirty procedures, such as draining an abscess or cleaning infected wounds. The room should be cleaned before use to the same standard as an operating theatre. Injections can be given in an operating theatre, but operating theatre protocols mean that it can take 15 minutes to prepare the patient and make the necessary checks, which may be an inefficient use of staff time.

You need a good light, so that you can see what you are doing. The patients should be lying flat on a comfortable couch or bed, which should be high enough for you to give the injections without bending over.

## Equipment

- Anti-VEGF drug
- Syringe – usually 1 ml as only a very small volume (0.05–0.1 ml) is injected
- Large bore needle – for drawing up the drug
- 30g needle – for giving injection
- 5% (aqueous) povidone iodine solution for disinfection of skin and conjunctiva
- Local anaesthetic drops
- Topical antibiotic drops
- Sterile cotton buds
- Sterile gloves
- Drapes
- Eyelid speculum
- Calliper or other measuring device

## Procedure

- Most patients will be understandably nervous about the prospect of having a needle stuck into their eye. You must provide sensible reassurance, and explain every step of the procedure so that your patient knows exactly what is going to happen next.
  **Figure 1:**
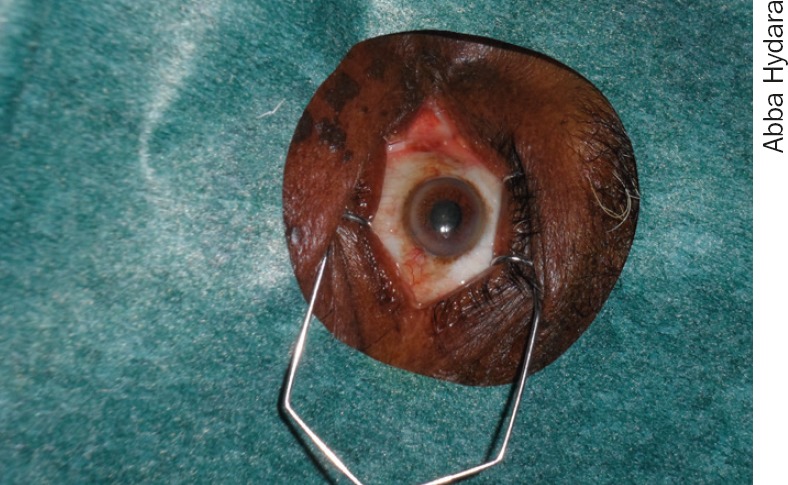

**Figure 2:**
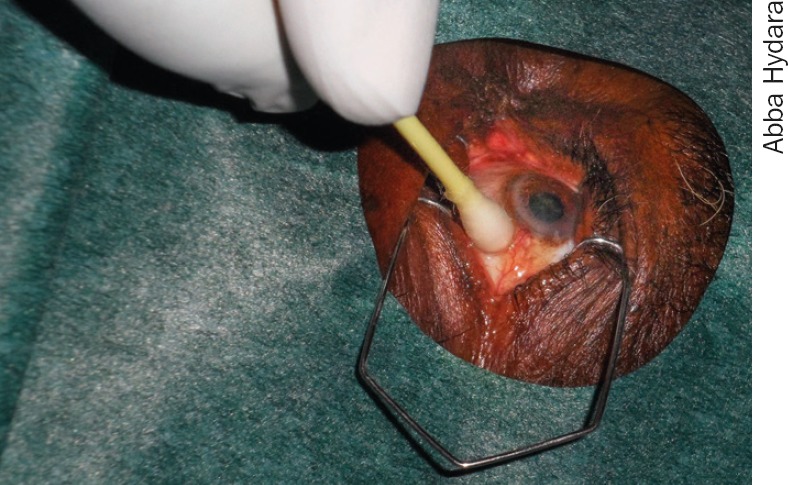

**Figure 3:**
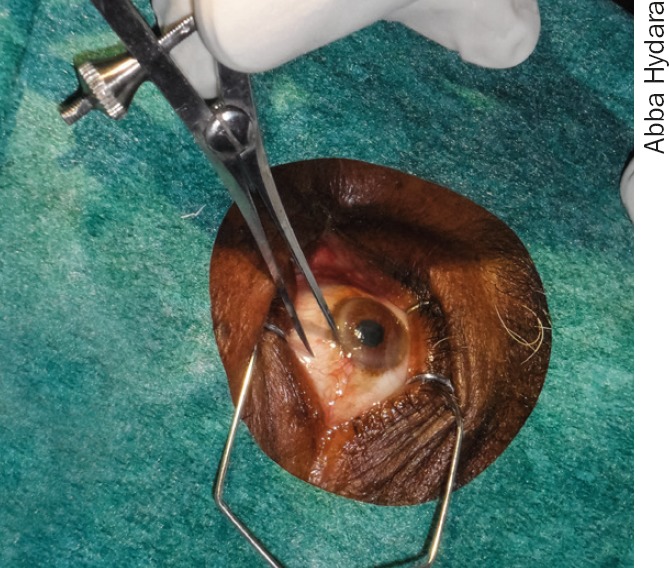

**Figure 4:**
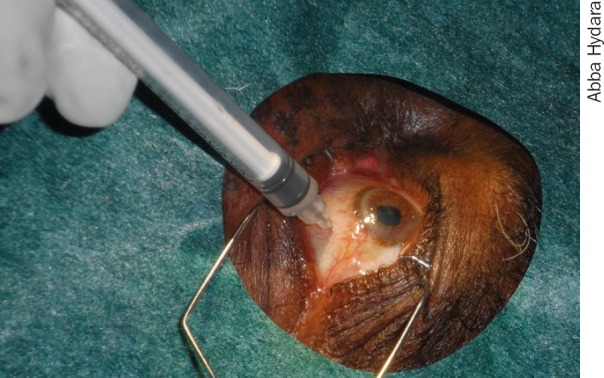
- Before you do anything to your patient, check the notes and prescription. You cannot see which eye needs to be treated so make sure that you are planning to inject the correct eye with the right drug. It is advisable to mark the eye to avoid any possible confusion.
- Once the patient is lying down comfortably, scrub your hands and put on sterile gloves. Some people will wear a sterile gown, but this is not essential.
- Instil some local anaesthetic drops. I usually put drops in both eyes, as the iodine solution is very irritant and may go into the other eye.
- The drops take a few minutes to work, so draw up the anti-VEGF while you are waiting. Use a sterile technique to draw up 0.1 ml into the 1 ml syringe, using a large bore needle. Empty the air from the syringe and fit the 30G needle on the syringe. Eject the surplus drug until there is 0.05ml left in the syringe.
- Use the 5% aqueous povidone iodine solution to clean and disinfect the eye to be injected. Wipe the skin around the eye and ensure that the solution goes into the conjunctival sac so that it also disinfects the surface of the eye. Leave the eye for a minute or so for the solution to work.
- Instil some topical antibiotic drops (the patient must complete the course).
- Dry the skin around the eye to remove excess povidone iodine and place a sterile drape over your patient's face so that only the eye to be treated is visible. Arrange the drape so that it does not obstruct your patient's breathing.
- Insert the speculum to hold the eye open (Figure 1).
- I usually place a swab soaked in local anaesthetic over the site of the injection and hold it in place for one minute (Figure 2)
- Using the measuring calliper or some other measuring device (Figure 3), measure a safe distance behind the limbus in the inferotemporal quadrant. In patients who have had cataract surgery, this is 3.5 mm. In patients who are phakic and still have their own lenses, it is 4 mm.
- Warn the patient that you are about to inject, insert the needle quickly and inject the drug (Figure 4), then remove the needle. Tell your patient it is all over!
- Instil more topical antibiotic drops, and check that the patient's vision is unaffected. Sometimes injection of even a small volume of fluid will cause a sharp rise in intraocular pressure. If this happens, the patients will notice a loss of vision. The best treatment is to do an immediate paracentesis to release aqueous from the anterior chamber. If this is not possible, however, ocular massage will usually lower the IOP. In patients who are at high risk from an elevation of IOP (e.g. people with severe glaucoma), it is sensible to do ocular massage before giving the injection.
- You should prescribe topical antibiotic drops for 4 days after the injection. You must also make follow-up arrangements, either for another injection, or to be reviewed in the clinic.

